# miR-23a-3p Regulates Runx2 to Inhibit the Proliferation and Metastasis of Oral Squamous Cell Carcinoma

**DOI:** 10.1155/2022/8719542

**Published:** 2022-03-18

**Authors:** Yu Ma, Jinbo Gao, Hongning Guo

**Affiliations:** Department of Stomatology, Tianjin Third Central Hospital, Tianjin 300170, China

## Abstract

**Objective:**

To investigate the effects of microRNA-23a (miR-23a-3p) and Runx2 on malignant progression of oral cancer cells and their possible molecular mechanisms.

**Methods:**

Fluorescence quantitative PCR (qPCR) was used to detect the expression of miR-23a-3p and Runx2 in human oral squamous cell carcinoma tissues and paracancerous tissues. The dual luciferase reporter assay was used to evaluate the targeted regulation of miR-23a-3p on Runx2. A subcutaneous xenograft model was established to investigate the tumor-suppressive effect of miR-23a-3p. Cells were transfected with miR-23a-3p mimics and negative control NC. CCK-8 assay, EDU assay, Transwell assay, and clone formation assay were used to detect malignant evolution of cells. Western blotting was used to detect the expression of Runx2, PTEN, and PI3K/Akt. The cells were simultaneously transfected with miR-23a-3p mimics and Runx2 to detect the malignant evolution of cells.

**Results:**

The expression of miR-23a-3p was downregulated in oral squamous cell carcinoma tissues, while the expression of Runx2 was upregulated. Overexpression of miR-23a-3p or inhibition of Runx2 inhibited the malignant progression of oral squamous cell carcinoma CAL-27 and TSCCA. Overexpression of miR-23a-3p inhibits the growth of oral cancer tumors. miR-23a-3p inhibits the PTEN/PI3K/Akt signaling pathway through Runx2. Overexpression of Runx2 reverses the tumor-suppressive effect of miR-23a-3p.

**Conclusion:**

miR-23a-3p can inhibit the PI3K/Akt signaling pathway by targeting Runx2 and inhibit the malignant evolution of oral cancer.

## 1. Introduction

OSCC is prone to local recurrence and distant metastasis. Therefore, early detection, diagnosis, and treatment have positive significance for improving the survival rate of OSCC [1]. The occurrence and development of OSCC involve many genes and proteins. Therefore, it is of great significance to screen out the molecular markers with high diagnostic efficiency for targeted therapy [2].

Abnormal expression of miRNA or changes in factors affecting miRNA generation will lead to the occurrence of some diseases such as tumors [3]. In microRNAs, miR-23a belongs to miR-23~24~27 clusters. miR-23a-3p is the mature chain of miR-23a [4]. miR-23a acts as a tumor inhibitor in osteosarcoma [5]. The overexpression of miR-23a reduces the proliferation of osteosarcoma cells and inhibits their migration and invasion. In addition, miR-23a inhibited epithelial-mesenchymal transformation (EMT) in endometrioid adenocarcinoma [6]. In contrast, miR-23a acts as an oncogene in gastric cancer. Reducing the expression level of miR-23a can inhibit the proliferation and metastasis of gastric cancer cells [7, 8]. However, the role of miR-23a-3p in oral squamous cell carcinoma need further study. Through TargetScan website, we predicted that RUNX2 might be the target gene of miR-23a. Therefore, we also studied the targeted regulatory relationship between RUNX2 and miR-23a. The role of RUNX2 in OSCC is also analyzed.

Runt-related transcription factors (RUNX) are one of the most important transcription factors in cell signaling. Runx plays a key role in development, cell proliferation, differentiation, and apoptosis. Recent studies have shown that RUNX is closely related to the occurrence and progression of tumors. The Runx family consists mainly of Runx1, Runx2, and Runx3 [9]. Runx family proteins interact with TGF-*β* signaling [10], Wnt signaling [11], Indian hedgehog signaling (IHH) [12], Notch signaling [13], RTK signaling [14], and many other key differentiation and development signaling pathways. Runx coordinates the transcription of different genes. Akech et al. [15] found that Runx2 was highly expressed in metastatic prostate cancer cells and could promote bone metastasis of cancer cells and osteolytic lesions. Meanwhile, high expression of Runx2 plays a role in breast cancer metastasis. Runx2 promotes bone metastasis of breast cancer through the ITGBL1-mediated TGF-*β* signaling pathway [16]. Runx2 is an independent prognostic marker in gastric cancer. Runx2 expression was associated with differentiation and invasion of gastric cancer. However, the role of Runx2 in OSCC needs to be further studied.

The objective of this study was to investigate the expression, function, and potential target genes of miR-23a-3p in oral squamous cell carcinoma. To explore the tumor inhibition mechanism of miR-23a-3p on oral squamous cell carcinoma.

## 2. Methods

### 2.1. Tissue and Clinical Information Collection of Oral Squamous Cell Carcinoma

Pathological specimens of 20 cases of OSCC treated in our hospital from March 2020 to January 2021 were collected for study. There were 13 males and 7 females in OSCC patients. The average age was 52.63 ± 8.96 years. In addition, oral mucosal epithelial tissue samples from 20 patients undergoing alveolar ridge plastic surgery in our hospital during the same period were collected as the control group. All the above pathological specimens were stored in liquid nitrogen immediately after removal. The following are the inclusion criteria: [1] all specimens that were confirmed histopathologically; [2] patients who did not receive radiotherapy, chemotherapy, or other related treatments before surgery; [3] first onset; and [4] patients who gave informed consent and had complete clinical data. The following are the exclusion criteria: [1] accompanied by the malignant tumor of other sites; [2] with infectious, toxic, or autoimmune diseases; and [3] with serious cardiovascular and cerebrovascular diseases and endocrine system diseases. All patients signed an informed consent form. This study was approved by the Ethics Committee of Tianjin Third Central Hospital.

### 2.2. Cell Culture

OSCC cell lines (SCC-9, Tca8113, TSCCA, and CAL-27) and normal oral keratinocyte (NHOK) cells were purchased from American Type Culture Collection (ATCC, Manassas, VA, USA). Then, they were inoculated in a 25 cm^2^ flask and DMEM medium containing 10% FBS and 1% penicillin, and streptomycin (Life Technologies, Rockville, MD, USA) was added. Immortalized oral epithelial cell lines were inoculated in a flask and cultured in SFM medium, then placed in an incubator (37°C, 5% CO_2_, and 95% water saturation) for routine culture. The logarithmic growth phase cells were used for the experiment.

### 2.3. Cell Transfection

A total of 1.5 × 10^5^ logarithmic growth cells were seeded into the six-well plate. miR-23a-3p inhibitor, miR-23a-3p mimics, and control were transfected into cells in different wells, respectively. miR-23a-3p inhibitor, miR-23a-3p mimics, and negative control sequences were diluted with 200 nmol/L miR-23a-3p inhibitor, miR-23a-3p mimics, and negative control sequences in double DMEM-free medium (without serum and double antibody) with 50 *μ*L, respectively. In addition, 2 *μ*L Lipofectamine 2000 was diluted into 50 *μ*L DMEM-free medium. Mix gently, and incubate at room temperature for 5 min. Then, diluted Lipofectamine 2000 was added into the diluted transfection sequence and mixed. Mix gently, and incubate at room temperature for 20 min. The complex was added to different holes and grooves, respectively, and 1.9 mL DMEM medium was added to each hole, and the amount of medium in each hole was 2 mL. The transfection concentration in the three holes and grooves was 100 nmol/L. At 37°C and 5% CO_2_, after 8 h in the incubator with 95% water saturation, the culture was replaced with 5% complete medium for another 40 h. Then, total RNA was extracted by RNAISO Reagent. Real-time fluorescence quantitative PCR was used for detection. Targeting-specific siRNA sequences of RUNX2 are as follows: the upstream primer: 5′-GAAGCUUGAUGAUAAATTAdTdT-3′, downstream primers: 5′-UUUAGAGUCAUCAAGCUUCTTCdTdT-3′. Interfering™ at a concentration of 10 nmol/L was used to transfect the cells in logarithmic growth phase, following the kit instructions. Runx2 siRNA was added to the cell culture medium at a final concentration of 40 nmol/L and transfected 48 h later for subsequent experiments.

### 2.4. qRT-PCR

Total RNA was extracted by Trizol method. RNA was reverse transcribed into cDNA with reference to the RNA reverse transcription kit. The reaction system was 20 *μ*L: 2 × SYBR Premix 10 *μ*L, H_2_O 8 *μ*L, CDNA1 *μ*L, and upstream and downstream primers 0.5 *μ*L each. The following is the reaction procedure: initial 95°C predenaturation for 5 m in, 95°C denaturation for 10 s, 60°C annealing for 30 s, 72°C for 2 min, 40 cycles. Extended at 72°C for 10 min. The upstream primer sequence of Runx2 is 5′-CCGGAATGCCTCTGCTGTTATGA-3′, and the downstream primer sequence is 5′-ACTGAGGCGGTCAGAGAACAAACT-3′. According to the 2^-*ΔΔ*CT^ algorithm, GADPH was used as the internal reference gene to calculate the mRNA expression. The miRNA expression was calculated using U6 as the reference gene.

### 2.5. Western Blot Test

The cleavage solution was added to fully cleavage the tissue. The reaction was carried out on ice for 5 min, then centrifuged at 12000 × g at 4°C for 20 min. Sample 50 *μ*g for each sample. The protein was transferred to the PVDF membrane by wet transfer method, with 100 V constant pressure transfer of 100 min. PBS-T solution with 2%BSA was closed for 2 h. Primary Runx2 (1 : 2000) antibody was diluted in proportion to the instructions and incubated overnight at 4°C. The membrane was washed with PBS-T solution, and the corresponding secondary antibodies were incubated at room temperature for 1 h. Finally, after the film was fully washed, ECL luminescent solution was used for exposure imaging. The optical density of the strips was analyzed by Image-Proplus 5.1 Image analysis software. The relative expression was expressed by the ratio of the optical density value of the corresponding protein to the internal reference GAPDH.

### 2.6. CCK-8

You take cells in the logarithmic phase. 3000 cells per well were seeded into 96-well plates. Each group has 3 duplicate holes. Cell proliferation activity was detected at 72 h. CCK-8 solution (1/10 dissolved in DMEM) was added and incubated at 37°C with 50 mL/L CO_2_ for 1 h. The absorbance (*A*) value at 450 nm was determined by enzyme-linked immunoassay. The cell proliferation curve was plotted with the culture time as the horizontal axis and the average value of A as the vertical axis. The experiment was repeated three times.

### 2.7. EdU Experiment

When the cell reaches 70% and above, the planking is carried out. Cell status and cell density were observed the next day under an inverted microscope. The cells reach 70%~80% when paving. According to the groups seeded in 96-well plate after transfection, 100 *μ*L EDU dye reagent was added to each well after cell attachment. Incubate in a decolorizing shaker for 30 min at room temperature and away from light. Add another 100 *μ*L Hoechst33342 to each well. Incubate in a decolorizing shaker for 30 min at room temperature and away from light. EDU uptake was observed under a fluorescence microscope.

### 2.8. Transwell Experiment

Cells were starved for 12 h before the experiment. Melt glue on ice in advance to a liquid state. Mix 150 *μ*L serum-free DMEM medium with 50 *μ*L matrix adhesive. Apply 30 *μ*L diluted matrix adhesive evenly to each compartment. The chamber was placed in a 24-well plate in an incubator for 30 min to solidify the glue. The cells in logarithmic growth phase were digested by trypsin and centrifuged to make the single cell suspension. Adjust the concentration to 1 × 10^9^/L. After the gel of the chamber solidified, each group of cells was set up with 2 duplicate holes, each hole was 3 × 10^5^ cells. The DMEM culture system without serum in the upper compartment was cultured 200 *μ*L per well. Add the appropriate volume of cell suspension. The lower chamber was added with 500 *μ*L complete DMEM medium. The 24-well plate was placed in an incubator at 37°C and 5% CO_2_ for 24 h. The culture medium in the upper compartment was discarded, and the cells that did not cross the membrane in the upper compartment were gently wiped with a cotton swab. 4% paraformaldehyde crystal violet was added for fixation and dyeing for 2 h. The upper chamber was cleaned by PBS for 3 times. Five visual fields were randomly selected from each hole to take pictures and count. The experiment was repeated for 3 times.

### 2.9. Clone Formation Experiment

The cells were digested into a single cell suspension. 3000 cells were seeded into the 6-well plate, and the cells were evenly dispersed, cultured at 37°C with 50 mL/L CO_2_ for more than 2 weeks. DMEM was frequently observed and replaced. When the cells were visible to the naked eye, the culture was terminated for staining. Discard the medium, and soak with PBS for 2 times. Add 2 mL 10 g/L glutaraldehyde to fix and 2 mL 5 g/L crystal violet stain. Rinse with tap water for several times; then soak for 15 min to remove staining solution and dry. The experiment was repeated for 3 times.

### 2.10. Subcutaneous Tumor Bearing Test

The animal experiment process is reviewed and approved by the Ethics Committee of Tianjin Third Central Hospital. Experimental animals were female BALB/C-nu nude mice aged from 4 to 6 weeks. All experimental animals were kept in SPF animal house and had normal diet. The average body weight was 20-22 g (purchased from the Institute of Experimental Animals, Chinese Academy of Medical Sciences). Single cell suspension (100 *μ*L) with a cell concentration of 5 × 10^7^/mL was extracted with a sterile 1 mL syringe and seeded subcutaneous into the left groin of 20 nude mice, respectively. Tumor cells were inoculated and subcutaneously formed in all nude mice. The rats were grouped according to the random comparison table, with 10 rats in each group. The long diameter (*A*) and wide diameter (*B*) of the tumor were measured once a day with a vernier caliper, and the tumor growth curve was plotted after continuous observation for 5 weeks. After the final tumor measurement, the animals were euthanized. Subcutaneous tumor-bearing specimens of nude mice were taken out and photographed. The following is the tumor volume calculation formula: *V* = (*AB*^2^)/2.

### 2.11. Double Luciferase Reporter Gene Assay

The TargetScan (http://www.targetscan.org/vert_71/) website database was used to predict the binding sites of miR-23a-3p and Runx2. 50 nmol/L of control mimics (the control vector for miR-23a-3p mimics) or miR-23a-3p mimics and 500 ng of 3′UTR-WT or 3′UTR-MUT were simultaneously transfected into cells. Fresh medium was replaced 24 h after transfection. After further culture for 24 h, the cells were lysed with lysate buffer. The activity of luciferin and luciferase was determined using dual luciferase report assay kits. Fluorescence intensity was measured using a Glomax®20/20 luminescence meter.

### 2.12. Statistical Analysis

SPSS16.0 statistical software was used for statistical analysis. Measurement data were expressed as mean ± standard deviation. One-way analysis of variance (ANOVA) followed by a Bonferroni post hoc test was used for comparison among groups. The unpaired two-tailed Student *t*-test was used for comparison between the two groups. *P* < 0.05 was considered statistically significant.

## 3. Results

The expression of miR-23a-3p was downregulated in oral squamous cell carcinoma tissues, while the expression of Runx2 was upregulated.

**Figure 1:**
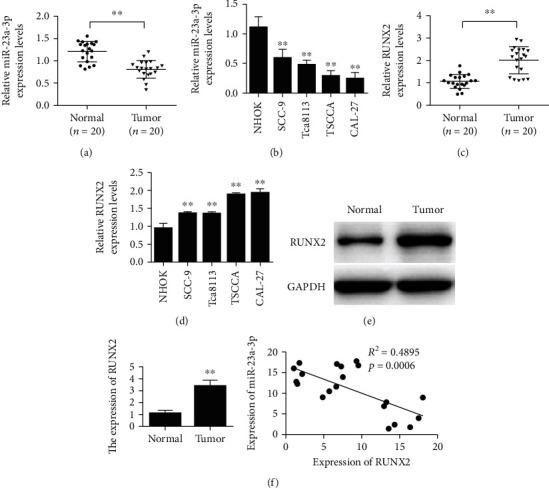
The expression of miR-23a-3p was downregulated in oral squamous cell carcinoma tissues, while the expression of Runx2 was upregulated. (a) Detection of miR-23a-3p expression level. (b) Detection of miR-23a-3p expression levels in normal oral keratinocytes (NHOK) and squamous cell carcinoma cells (CAL-27, TSCCA, TCA8113, and SCC-9). (c) Runx2 is upregulated in oral squamous cell carcinoma by qRT-PCR. (d) Runx2 expression was upregulated in human oral keratinocytes (NHOK) and squamous cell cells (CAL-27, TSCCA, TCA8113, and SCC-9). (e) Runx2 is upregulated in oral squamous cell carcinoma by Western blot. (f) Detection of correlation between miR-23a-3p and Runx2 coexpression. ^∗∗^*P* < 0.01.

The qPCR results showed that the expression of miR-23a-3p was downregulated in oral squamous cell carcinoma tissues compared with the control group (Figure 1(a)). The expression level of miR-23a-3p in normal oral keratinocytes (NHOK) was significantly higher than that of squamous cell carcinoma cells (CAL-27, TSCCA, TCA8113, and SCC-9) (Figure 1(b)). QPCR results showed that Runx2 expression was upregulated in oral squamous cell carcinoma (Figure 1(c)). Runx2 expression was upregulated in squamous cell carcinoma cells (CAL-27, TSCCA, TCA8113, and SCC-9) compared with normal oral keratinocytes (NHOK) (Figure 1(d)). Western blot results showed that Runx2 expression was upregulated in oral squamous cell carcinoma (Figure 1(e)). The coexpression correlation results showed that there was a negative coexpression correlation between miR-23a-3p and Runx2 in oral squamous cell carcinoma (Figure 1(f)).

### 3.1. miR-23a-3p Inhibited the Malignant Progression of OSCC

**Figure 2:**
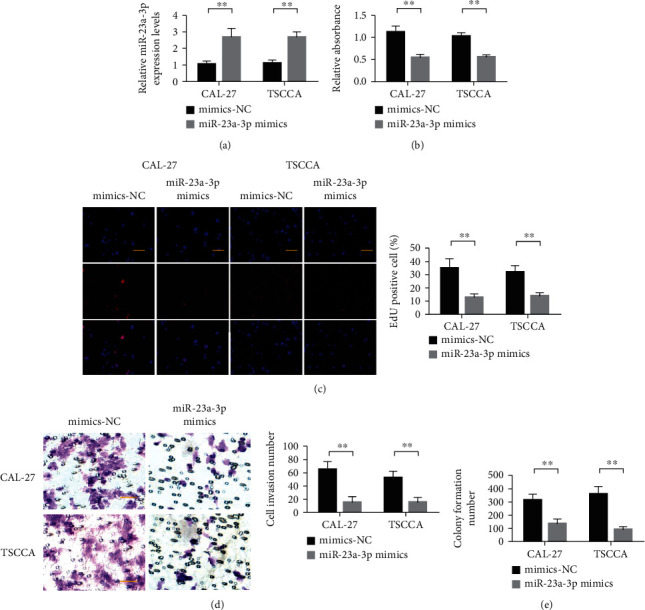
Overexpression of miR-23a-3p inhibited the malignant progression of CAL-27 and TSCCA in oral squamous cell carcinoma. (a) Detection of miR-23a-3p expression level efficiency in CAL-27 and TSCCA cells. (b) CCK-8 detected the proliferation of CAL-27 and TSCCA cells. (c) EDU was used to detect the proliferation of CAL-27 and TSCCA cells. (d) Transwell detection of CAL-27 and TSCCA cells. (e) Detection of clonal formation ability of CAL-27 and TSCCA cells. ^∗∗^*P* < 0.01.

We further examined the effects of miR-23a-3p on the proliferation, invasion, and clonogenesis of oral squamous cell carcinoma lines. Detection results of miR-23a-3p expression efficiency in CAL-27 and TSCCA cells showed that miR-23a-3p mimics could upregulate the expression level of miR-23a-3p (Figure 2(a)). Through CCK-8 experiment and EDU experiment, we found that the proliferation ability of CAL-27 and TSCCA cells overexpressing miR-23a-3p was significantly decreased compared with the control cells (Figures 2(b) and 2(c)). Through cell invasion assay, we found that overexpression of miR-23a-3p inhibited the invasion ability of CAL-27 and TSCCA cells (Figure 2(d)). We examined the ability of cells to grow unanchored after overexpression of miR-23a-3p by colony formation assay. The results showed that after the overexpression of miR-23a-3p, the number of colonies formed by CAL-27 and TSCCA cells was significantly decreased (Figure 2(e)). These results indicate that miR-23a-3p can reduce the malignant biological behavior of tumor cells and then affect the invasion and metastasis ability of tumor cells.

### 3.2. Overexpression of miR-23a-3p by Lentivirus Inhibits the Growth of Oral Cancer Tumors

**Figure 3:**
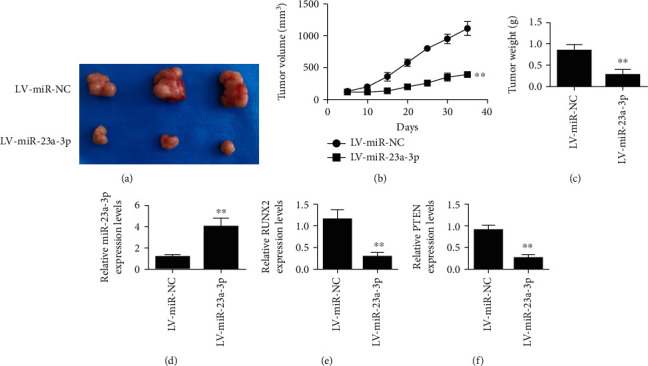
Overexpression of miR-23a-3p by lentivirus inhibited the growth of oral cancer tumor. (a) Representative images of tumors in the control group and the transfection group. (b) Tumor volume of miR-23a-3p-transfected CAL-27 cells was detected. (c) Tumor weight of miR-23a-3p-transfected CAL-27 cells was detected. (d) The expression level of miR-23a-3p in tumor tissues after tumor bearing of miR-23a-3p-transfected CAL-27 cells was detected. (e). The expression of Runx2 in tumor tissues of miR-23a-3p-transfected CAL-27 cells after tumor bearing was detected. (f) The expression level of PTEN in tumor tissues of miR-23a-3p-transfected CAL-27 cells after bearing tumor was detected. ^∗∗^*P* < 0.01.

Results of experiments in tumor-bearing animals showed that the overexpression of miR-23a-3p by lentivirus reduced the proliferation rate and tumor volume of oral squamous cell carcinoma cells (Figures 3(a) and 3(b)). Tumor weight was also decreased after overexpression of miR-23a-3p (Figure 3(c)). QRT-PCR was used to detect the expression of miR-23a-3p in tumor-bearing cells of CAL-27 transfected with miR-23a-3p. The results showed that the expression level of miR-23a-3p was increased in the transfection group (Figure 3(d)). The expression of Runx2 in tumor tissues was decreased after miR-23a-3p-transfected CAL-27 cells were tumorigenic (Figure 3(e)). In addition, miR-23a-3p-transfected CAL-27 cells bearing tumor also reduced the expression of PTEN in tumor tissues (Figure 3(f)).

### 3.3. Runx2 Knockdown Inhibited the Malignant Progression of Bladder Cells

**Figure 4:**
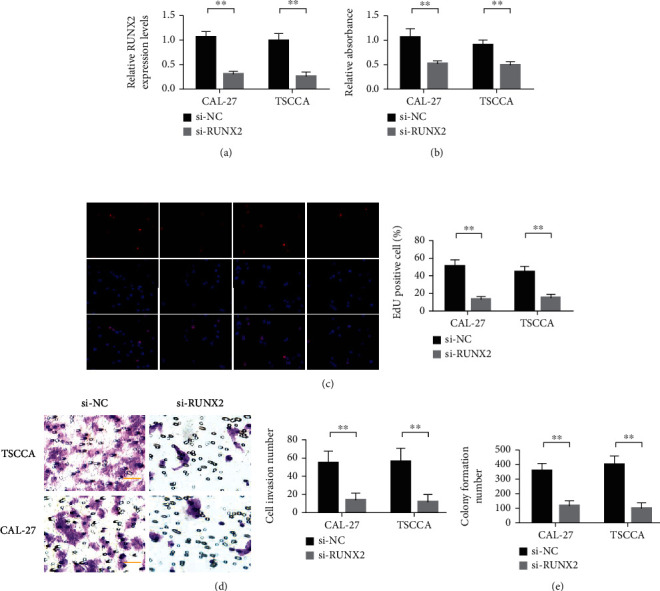
Runx2 knockdown inhibited malignant progression of bladder cells. (a) Detection of Runx2 expression in CAL-27 and TSCCA cells. (b) CCK-8 detected the proliferation of CAL-27 and TSCCA cells. (c) EDU was used to detect the proliferation of CAL-27 and TSCCA cells. (d) Transwell detection of CAL-27 and TSCCA cells. (e) Detection of clonal formation ability of CAL-27 and TSCCA cells. ^∗∗^*P* < 0.01.

The results of transfection efficiency test showed that siRNA Runx2 could reduce the expression of Runx2 in CAL-27 and TSCCA cells (Figure 4(a)). CCK-8 and EDU experiments showed that the difference between the Runx2 siRNA group and the control group was statistically significant (Figures 4(b) and 4(c)). These results indicated that Runx2 knockdown had a significant inhibitory effect on cell growth. Transwell assay and clone formation assay further demonstrated that Runx2 knockdown inhibited the invasion and clone formation of CAL-27 and TSCCA cells (Figures 4(d) and 4(e)).

### 3.4. miR-23a-3p-Targeted Runx2 Binding

**Figure 5:**
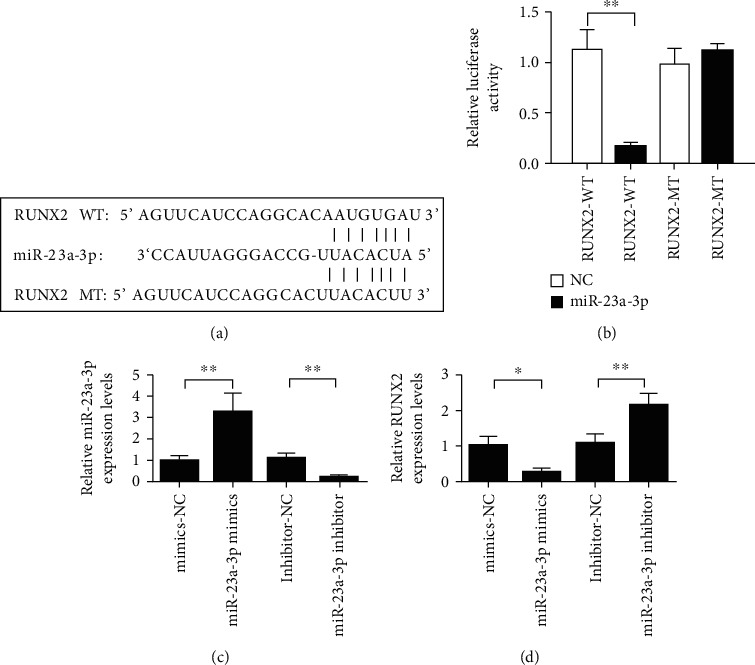
miR-23a-3p targeting binding Runx2. (a) Information diagram of the binding sites of miR-23a-3p and Runx2. (b) Dual-luciferase reporter genes demonstrate the binding of miR-23a-3p to Runx2. (c) Detection of transfection efficiency of miR-23a-3p. (d) Overexpression of miR-23a-3p inhibited Runx2, and inhibition of miR-23a-3p upregulated Runx2. ^∗^*P* < 0.05 and^∗∗^*P* < 0.01.

Through bioinformatics prediction, we found that Runx2 was the target gene of miR-23a-3p (Figure 5(a)). Subsequently, the binding of miR-23a-3p to Runx2 was verified by dual luciferase assay. First, we constructed psi-Runx2 wild-type and mutant vectors and synthesized psi-Runx2 wild-type or mutant vectors. The results showed that miR-23a-3p inhibited luciferase activity in the 3′UTR of wild miR-23a-3p. However, this inhibition was prevented in the 3′UTR of mutant Runx2. It was shown that miR-23a-3p could directly bind to the 3′UTR of Runx2 (Figure 5(b)). Overexpression of miR-23a-3p inhibited Runx2 at the level of CAL-27 cells. In contrast, decreasing the expression of miR-23a-3p can upregulate Runx2 (Figures 5(c) and 5(d)).

### 3.5. miR-23a-3p Inhibited the PTEN/PI3K/Akt Signaling Pathway through Runx2

**Figure 6:**
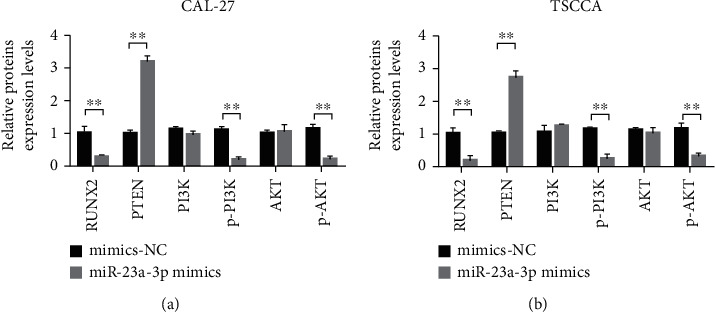
miR-23a-3p inhibited the PTEN/PI3K/Akt signaling pathway through Runx2. (a) RT-qPCR was used to detect the changes of mRNA expression in CAL-27 cells of each group after different treatments. (b) RT-qPCR was used to detect the changes of mRNA expression in TSCCA cells of each group after different treatments. ^∗∗^*P* < 0.01.

RT-qPCR results showed that miR-23a-3p overexpression inhibited the PTEN/PI3K/Akt signaling pathway in CAL-27 and TSCCA cells. Compared with that of the mimics-NC group, the overexpression of miR-23a-3p reduced the expression of Runx2 and PTEN. In CAL-27 and TSCCA cells, overexpression of miR-23a-3p had no effect on the total expression of PI3K and AKT. However, it can inhibit the expression of p-PI3K and p-Akt (Figures 6(a) and 6(b)).

### 3.6. Overexpression of Runx2 Reversed the Tumor-Suppressive Effect of miR-23a-3p

**Figure 7:**
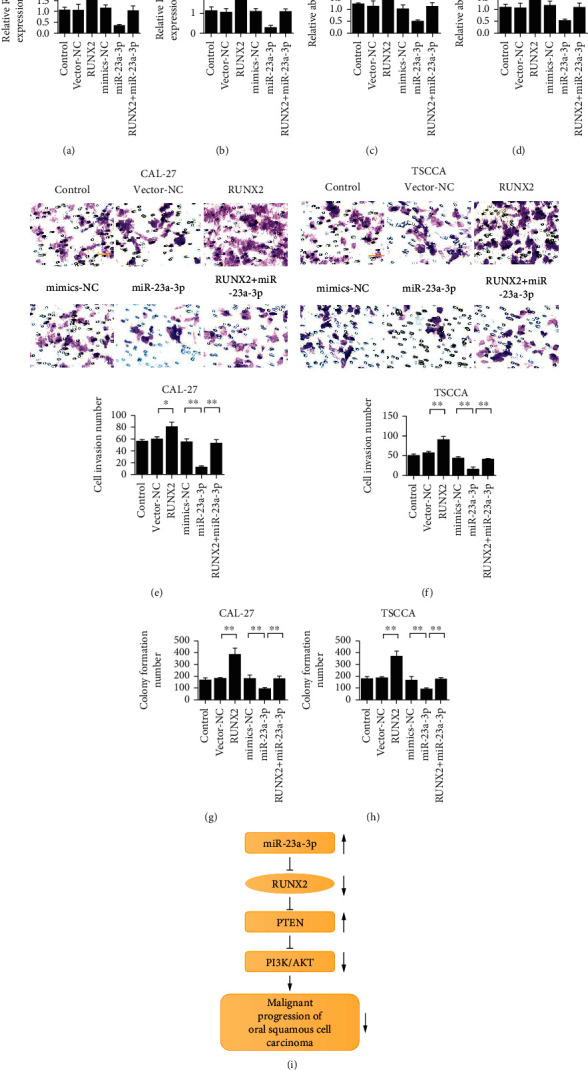
Overexpression of Runx2 reverses the tumor-suppressive effect of miR-23a-3p. (a) Detection of Runx2 expression in CAL-27 cells. (b) Detection of Runx2 expression in TSCCA cells. (c) Proliferation rate of CAL-27 cells. (d) Proliferation rate of TSCCA cells. (e) Transwell detection of CAL-27 cells. (f) Transwell detection of TSCCA cells. (g). Detection of clonal formation ability of CAL-27 cells. (h) Detection of TSCCA cell clone formation ability. (i) Full-text mechanism diagrams. ^∗^*P* < 0.05 and^∗∗^*P* < 0.01.

To further verify the targeted binding relationship between miR-23a-3p and Runx2 and its role in oral squamous cell carcinoma, we simultaneously transfected miR-23a-3p mimics and Runx2 overexpression plasmids and detected the changes in cells by CCK-8, Transwell, and clone formation assay. Overexpression of miR-23a-3p inhibited the expression of Runx2. Meanwhile, transfection of Runx2 overexpression plasmid reversed the inhibitory effect of miR-23a-3p (Figures 7(a) and 7(b)). Compared with the control group, the proliferation ability of CAL-27 and TSCCA cells in the overexpressed miR-23a-3p group was decreased, with statistically significant differences. Compared with the miR-23a-3p group, the invaded cells transfected simultaneously with Runx2 and miR-23a-3p increased their proliferation ability (Figures 7(c) and 7(d)). Compared with that in the control group, the number of cells passing through the matrix gel of CAL-27 and TSCCA cells in the overexpressed miR-23a-3p group was significantly reduced. Compared with the miR-23a-3p group, the number of invaded cells transfected with both Runx2 and miR-23a-3p was increased (Figures 7(e) and 7(f)). The results of the clone formation experiment showed that the number of clones was reduced in the overexpressed miR-23a-3p group compared with the control group. Compared with the miR-23a-3p group, the number of clone cells transfected simultaneously with Runx2 and miR-23a-3p was increased (Figures 7(g) and 7(h)).  Figure 7(i) shows the full-text mechanism diagram of this study.

## 4. Discussion

About 50% of miRNAs are located in regions where currently known oncogenes are often amplified or deleted [17]. Therefore, the abnormal expression of some miRNAs is closely related to tumorigenesis [18]. miRNAs can regulate gene expression in the human genome by inducing mRNA degradation or inhibiting gene translation by targeting specific sites in the 3′-UTR region of mRNA. The expression level of miR-23a-3p was downregulated in oral squamous cell carcinoma, suggesting that miR-23a-3p may be a tumor suppressor gene of oral squamous cell carcinoma. Recently, numerous studies have shown that miR-23a-3p is upregulated in a variety of cancers and is closely related to the development of cancer. miR-23a-3p is expected to become a novel biomarker and therapeutic target [19–21]. It has been reported that the expression level of miR-23a-3p in serum samples from patients with colon cancer is higher than that of healthy donors. However, higher miR-23a-3p may be involved in the disease process at an early stage of cancer development. It has been experimentally confirmed that transfer suppressor 1 (MTSS1) is the target gene of miR-23a-3p. miR-23a-3p may affect tumor invasion ability through MTSS1 [22]. Another experiment suggested that miR-23a-3p was highly expressed in esophageal squamous cell carcinoma and was closely related to tumor differentiation. miR-23a-3p may play an important role in the microenvironment of esophageal cancer [23].

In this study, in order to determine the expression level of miR-23a-3p, we used oral squamous cell carcinoma tissue samples for qRT-PCR analysis. The results of this study showed that miR-23a expression level was 0.81 ± 0.20 in paracancer tissues, which was significantly lower than that in tumor tissues (1.21 ± 0.23). Therefore, we speculated that miR-23a-3p has a tumor-suppressive effect in oral squamous cell carcinoma. The downregulation of miR-23a expression in glioma cell lines suggests that miR-23a may be a tumor suppressor gene of oral squamous cell carcinoma. Subsequent cell experimental data showed that overexpression of miR-23a-3p inhibited cell growth and reduced cell invasion and clone formation ability. Bioinformatics studies have shown that Runx2 is a potential target gene of miR-23a-3p, so we confirmed that miR-23a-3p can indeed target the 3′UTR of Runx2 binding mRNA through dual luciferase assay.

To date, only a few targets of miR-23a have been experimentally validated. In this study, the double luciferase reporter assay confirmed that miR-23a inhibited the luciferase activity of wild-type RUNX2 3′UTR reporter gene vector. The luciferase activity of mutant RUNX2 3′UTR was not affected. This suggests a targeted regulatory relationship between RUNX2 and miR-23a.

Runx2 is a transcription factor belonging to the Runx gene family. Runx2 has a conserved runt DNA-binding domain that encodes a protein homologous to Drosophila [24]. Initially, Runx2 was thought to be a major regulator of bone development [25]. To date, a large body of evidence suggests that Runx2 is closely associated with tumor metastasis [26]. Runx2 is highly expressed in breast cancer cell lines and breast primary tissues that are more prone to bone metastasis [16, 27]. There is evidence that Runx2 can regulate the expression of genes and matrix metalloproteinases related to bone metastasis in breast cancer cells, promote breast cancer to degrade the cellular matrix, destroy the basement membrane and invade blood vessels or lymphatic vessels, and facilitate the colonization of tumor cells at distant metastatic sites [28, 29]. In addition, Runx2 has been shown to promote proliferation and migration of esophageal cancer cells. Runx2 knockdown leads to cell proliferation arrest and apoptosis in esophageal carcinoma cells [30]. This study suggests a targeted regulatory relationship between Runx2 and miR-23a-3p. This study further examined the effect of Runx2 knockdown on the biological behavior of oral squamous cell carcinoma. CCK-8 assay and plate clone formation assay indicated that downregulation of Runx2 expression inhibited the growth and viability of oral squamous cell carcinoma cells. These results suggest that Runx2 has a malignant potential in promoting the growth and survival of cancer cells in colon cancer. Experimental methods of tumor cell migration and invasion showed that the number of oral squamous cells in the Runx2 si-RNA group crossed the stromal gel significantly less than that in the stable expression control group.

## 5. Conclusion

The overexpression of miR-23a-3p can significantly inhibit the proliferation, invasion, and clonogenesis of oral squamous cell carcinoma cells. Mechanism studies suggest that the tumor-suppressive effect of miR-23a-3p may inhibit the malignant progression of oral squamous cell carcinoma by inhibiting the Runx2 regulation of PTEN/PI3K/Akt signaling pathway. In this study, we elucidated the molecular mechanism of miR-23a-3p's inhibition of microRNAs in oral squamous cell carcinoma. This study provides a new target and theoretical basis for oral squamous cell carcinoma.

## Data Availability

The analyzed datasets generated during the study are available from the corresponding author on reasonable request.
